# Forest resources of nations in relation to human well-being

**DOI:** 10.1371/journal.pone.0196248

**Published:** 2018-05-14

**Authors:** Pekka E. Kauppi, Vilma Sandström, Antti Lipponen

**Affiliations:** 1 Ecosystems and Environment Research Programme, Faculty of Biological and Environmental Sciences and Helsinki Institute of Sustainability Science (HELSUS), University of Helsinki, Helsinki, Finland; 2 Finnish Meteorological Institute, Atmospheric Research Centre of Eastern Finland, Kuopio, Finland; Natural Resources Canada, CANADA

## Abstract

A universal turnaround has been detected in many countries of the World from shrinking to expanding forests. The forest area of western Europe expanded already in the 19^th^ century. Such early trends of forest resources cannot be associated with the rapid rise of atmospheric carbon dioxide nor with the anthropogenic climate change, which have taken place since the mid 20^th^ century. Modern, most recent spatial patterns of forest expansions and contractions do not correlate with the geography of climate trends nor with dry versus moist areas. Instead, the forest resources trends of nations correlate positively with UNDP Human Development Index. This indicates that forest resources of nations have improved along with progress in human well-being. Highly developed countries apply modern agricultural methods on good farmlands and abandon marginal lands, which become available for forest expansion. Developed countries invest in sustainable programs of forest management and nature protection. Our findings are significant for predicting the future of the terrestrial carbon sink. They suggest that the large sink of carbon recently observed in forests of the World will persist, if the well-being of people continues to improve. However, despite the positive trends in domestic forests, developed nations increasingly outsource their biomass needs abroad through international trade, and all nations rely on unsustainable energy use and wasteful patterns of material consumption.

## Introduction

A large and persistent sink of carbon has been detected in forests of the World [1]. Terrestrial ecosystems including forests have become increasingly green [2]. The flux of carbon from the atmosphere into land ecosystems and oceans has increased [1,3]. However, there is large variation between forested nations of the World in terms of the rate of change of their national forest resources and even the sign of this change [4]. The forested area and/or forest biomass expand in a large number of countries while deforestation and forest degradation prevail in all too many countries with profound negative impacts on biodiversity, carbon stocks and the ecosystem services in general [5,6]. From the perspective of policy development, it is very important to understand, why forest ecosystems of World nations respond to global environment changes in such a surprisingly diverse fashion.

Scientific analyses have applied a number of attributes for quantifying forest trends. Remote sensing instruments have observed ´greening´ as the growing season integrated LAI, indicating the vigor of plant canopies (leaves and chlorophyll) [2]. Concepts `land area covered by forests´ or ´forest cover´ have been used in deforestation research detecting land conversion from forest to non-forest [7]. The concepts ´growing stock volume´ or ´plant biomass´ indicate the volume or mass of plant material in forests. Approximately one half of dry plant material consists of carbon and, therefore, ´biomass´ (B) has often been converted to ´carbon stock´ (C); C = ½B [8].

The history of change in forest cover and forest resources has been investigated applying such attributes. In some areas, land use patterns have not changed. As one extreme, there has been little changes in land use in the wilderness of Siberia, interior Congo or boreal regions of Canada. As another extreme, most of the original forests of Iceland and Azerbaijan were converted to rangelands and other uses long time ago. As reforestation is now being implemented, forest resources of such countries could easily double in a short time. Most countries lie between these two extremes. A fraction of the original forest land is preserved, while another significant fraction has been converted to agriculture, settlements and infrastructure [9].

Keenan et al. [10] summarized the latest FAO data on the dynamics of the global forest area reporting that forest area expanded in Europe, North America, the Caribbean, East Asia, and Western Central Asia, but decreased in Central America, South America, South and Southeast Asia, and all three regions of Africa. In 2010–2015, net tropical forest loss was dominated in South America by Brazil, in Asia by Indonesia, and in Africa by Nigeria. Between 1990 and 2015 according to [10] thirteen tropical countries may have either experienced national transitions from net forest loss to net forest gain, or continued along the path of forest expansion that follows such transitions.

Multiple global models suggest that in recent decades CO_2_ fertilization explains most of the global greening trends [2]. These models do not contain land use change nor land management explicitly as independent variables.

### Forest transition

Historically, land management has affected the vast majority of terrestrial ecosystems for many centuries long before the CO_2_ concentration of the atmosphere started to rise [11,12]. The general long term trajectory of forested area of nations follows a universal pattern from shrinking to expanding forest area. The turnaround from diminishing to increasing forests is called `forest transition`[13,14]. In a large number of countries, a shift has occurred from net deforestation to net reforestation [13]. Most tropical countries have not yet reached forest transition [15]. However, the rate of total net forest loss halved from 7.3 Mha yr-^1^ in the 1990s to 3.3 Mha yr^-1^ in 2010 to 2015 [4] and [10].

Two mechanisms operate as drivers of forest transition sometimes counteracting and sometimes reinforcing one another. First, urbanization and non-farm jobs pull farmers off the rural lands. The fields, often up in the mountains or otherwise located in margins of agriculture, return to forests. Second, a scarcity of wood-derived products attracts governments and landowners actively to create new forests [7]. Losses of forest area have occurred in moist areas such as Brazil and Indonesia, while gains of forest area have been detected in dry areas of Central Asia. Humidity of climate does not correlate with the global fingerprint of forest change [13,16].

As nations over time become wealthier and better organized, objectives and practices of land management change profoundly. Mather and Needle [17] mention the following five drivers of development, which lead to forest transitions and, further, to an expansion of forested lands. 1) Farmers adjust agriculture to land quality that is; they learn to select most suitable lands for agriculture and to abandon marginal farmlands. Areas favored by climate, soil and slope account for a growing proportion of agricultural production. 2) Evolution from a subsistence regime to a market economy further concentrates farming to the best lands, relaxing the pressure on other lands, which then become available for forest expansion. Under subsistence regime, the scale of farming is local. Under market regimes, the scale is provincial, national or even global thus amplifying the relative advantage of the very best farmlands. 3) Rural exodus promotes the transition from subsistence farming to the market regimes. 4) Agricultural technologies and the yields improve thereby relaxing the demand of clearing new agricultural land. Improved technologies provide little benefit on marginal lands while the benefit is great on the best lands. 5) As nations evolve, the development of railways and other modes of communication make market orientation increasingly feasible. Moreover, the demand for agricultural land depends on food waste during farming and harvesting. Storage facilities, transportation, processing, chilling chains and consumer behavior have an impact on how much food becomes wasted [18]. The demand for agricultural land is relaxed, if the losses can be reduced. At present, fuel wood is still used for cooking in a large scale in many developing regions of the World. As it is replaced by fossil fuels or by an expansion of the electricity grid, fuel wood harvests decrease. Drivers, which trigger forest transitions, also affect the opportunities for reforestation. Easy access to, and market availability of recently abandoned farmlands attracts active cultivation of industrial or domestic round wood.

While implementation of market economies favors new forest transitions, population growth and the increasing demand for food restrict land abandonment and forest expansion. However, in developed economies the forces of technological advance and agricultural intensification have outweighed the impacts of population growth and improving diets [19]. Therefore, we observe a global diffusion of forest transitions [8,13].

The focus of this research is on the impact of social, economic and technological trends on forest transitions and the rate of change of forest resources. Earlier research has shown that environmental changes have affected forests and contributed to the global greening. Our new research asks the following questions: Where and when did the national and regional forest resources shrink and where and when did they expand? During the period 1990 to 2015, how did the forest resources of nations change, and how can the observed changes be understood? Can the environmental changes such as CO_2_ fertilization and climatic warming satisfactorily explain the observed time evolution and geographical fingerprint of the positive and negative forest trends? We test the following hypothesis: Forest resources of the World expand because forest ecosystems respond primarily to environmental changes; while social, economic and technological impacts on land use and land cover play only a marginal role in determining the global temporal and spatial patterns of shrinking vs. expanding forests.

## Timing and present distribution of transitions

**Fig 1:**
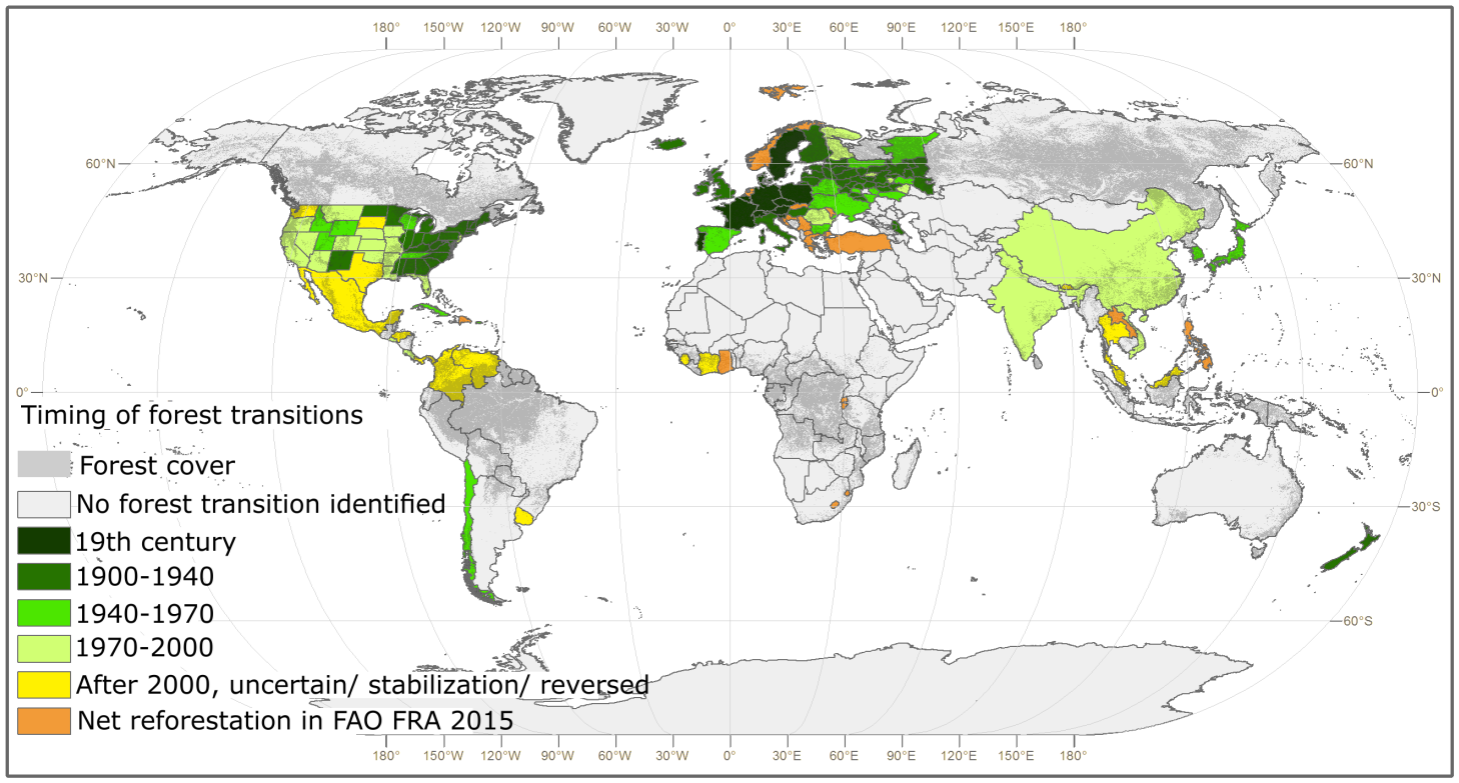
Timing of forest transitions, updated from ref. [13] to correspond the latest information in 2018. For references, see S1 Supporting Information.

The forest transition (= turnaround from shrinking to expanding forest area) has taken place in a large number of World countries and regions (Fig 1).

The geographical diffusion of forest transitions has correlated with the historical switch from subsistence farming to market oriented agriculture. First, forest transitions took place in Western and Central Europe and eastern United States, then in Northern and Eastern Europe, Japan and New Zealand, in the last fifty years in Chile and China and, finally, quite recently also in some subtropical and tropical countries of Latin America, Africa and the Far East. Compared to the earlier version of this map as of 2011 [13], increasing detail is here shown within the area of the former Soviet Union. Transitions in Latin America and Africa are uncertain and perhaps reversible. Africa is the continent with a great risk of further losses of forest ecosystems, because a majority of the 55 African countries has not reported forest transition.

A forest transition was reported for India during 1970–2000 (Fig 1). The population of India grew from 555 to 1,231 million between 1970 and 2010, respectively. Despite the needs of such a large and fast growing population the forests of the country ceased to shrink. This example shows how forests in the modern World can coexist with intensive population growth.

The vast and remote forests of Canada and Siberia do not appear on the forest transition map, notably because only small tracts of these sparsely populated and pristine forests were converted to non-forest uses in the first place. Regional detail of forest transitions within large countries like Brazil, China and India remains as an interesting challenge for future research.

## Correlation of forest change with climate warming, national income and human well-being

The long-term diffusion of forest transitions (Fig 1) shows that forests expanded in many countries in the 19^th^ and early 20^th^ century, when the global climate was relatively stable and the atmospheric CO_2_ concentration was near the pre-industrial level. In order to analyze the geography of more recent forest trends we compiled data from 103 countries representing 75% of the global forest area, and related them to economic and social attributes, see S1 Supporting Information for the list.

During 1990–2015, the forest growing stock (GS) decreased on average by 0.72% annually in low-income countries but hardly changed at all in lower-middle income countries, see S1 Supporting Information for the definition of GS. In higher-middle income and high-income countries the growing stock increased annually by 0.5 and 1.31%, respectively (Table 1). Global maps showing the changes from 1990 to 2015 in forest area and the growing stock are shown in S1 Supporting Information.

**Table 1:** Mean annual change in forest growing stock (Δ GS) in countries, 1990–2015, in relation to income level.

	Low-income (LI) (n = 22)	Lower-middle income (LMI) (n = 27)	Higher-middle income (HMI) (n = 29)	High-income (HI) (n = 25)
Δ GS (% per year)	-0.72	-0.29	0.50	1.31
st deviation of Δ GS	0.91	1.32	1.16	0.95

Statistically significant differences in Δ GS were detected between country groups as follows: HMI vs. HI (p = 0.002), LMI vs. HI (p = 3.6e-6), LI vs. HI (p = 2.9e-8), LMI vs. HMI (p = 0.02), and LI vs. HMI (p = 1.6e-4) using Mann–Whitney U-test, pairwise. The difference LI vs LMI was not statistically significant. The complete data by countries are shown in S1 Supporting Information.

**Fig 2:**
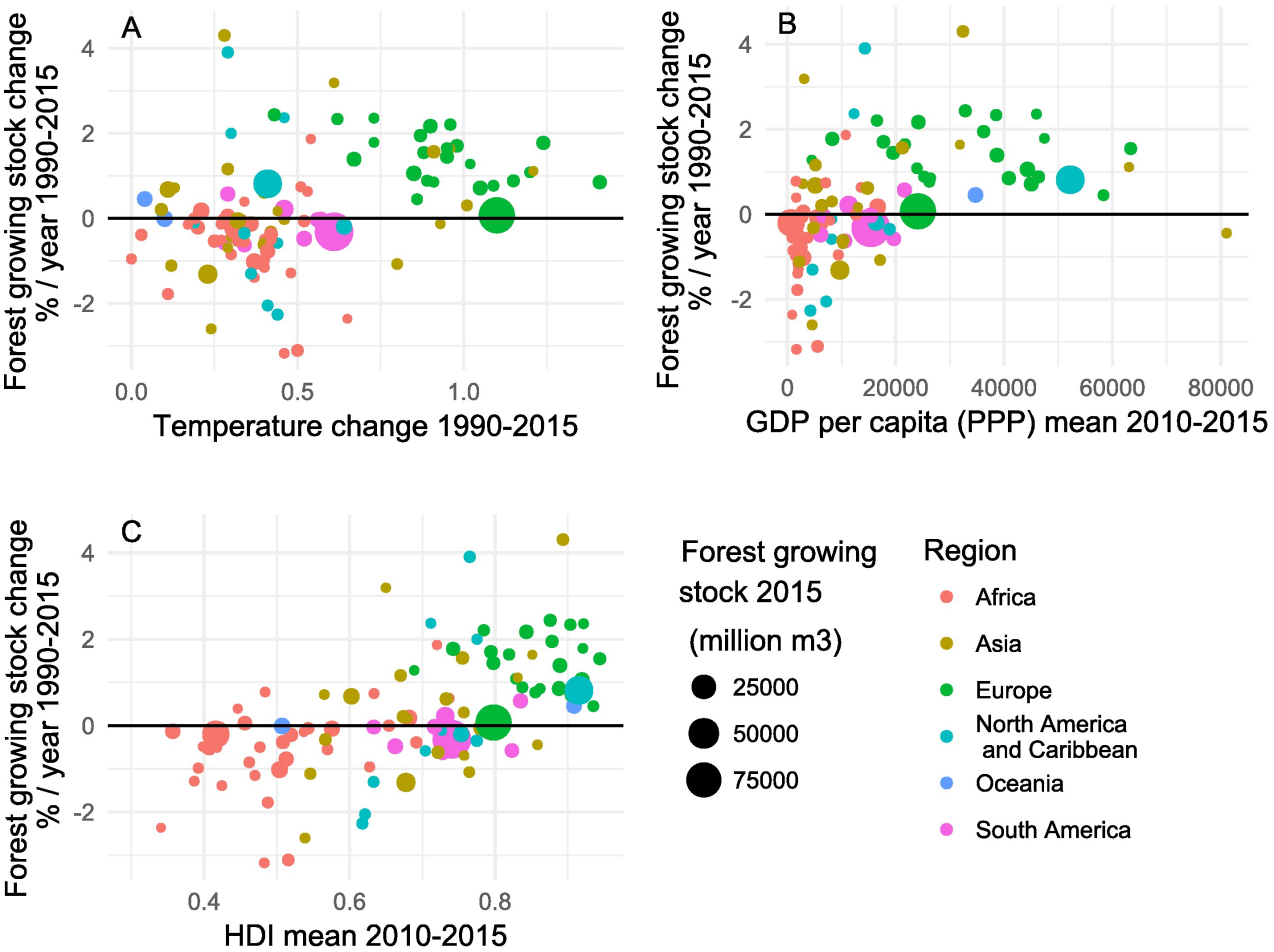
Δ GS in nations 1990–2015 as a function of A) temperature change (degrees C); B) average income (GDP per capita per year, and C) Human Development Index (HDI). Dot size shows the growing stock volume in a country in 2015 and dot color indicates the region.

Warming rather than cooling was typically observed in these 103 nations. A trend analysis for the temperature data for period 1985–2015 indicated statistically significant increasing temperature trends in most of the countries (p-value < 0.05). There was no country with a statistically significant decreasing trend of the mean temperature. A video by Antti Lipponen is included in S1 Supporting Information showing global temperature trends over a long time perspective. However, Δ GS did not correlate with temperature change with an exception in Europe, where comparatively large warming was detected in combination with expanding stocks of growing stock and hence carbon sequestration (Fig 2A). Elsewhere, the change of growing stock was virtually unrelated to the rate of climate warming.

Earlier research suggested a threshold at 4,600 USD (of 2003), income per capita, above which the growing stock did not decrease [8]. A similar result was reported assessing the rate of change of forest cover [20]. However, in this new analysis we could not reproduce such a clear threshold for the period 1990–2015; or a similar threshold is much higher at around 20,000 dollars income per capita (of 2010–2015; Fig 2B). This could partly reflect the impact of expanding international trade on the global land use [21]. Net exporters of agricultural products convert forests to arable land, while concurrently their national income level improves. As in [8] deviating forest trends in low-income countries and a consistent positive trend in high-income countries were here reproduced, with Brunei as the sole exception (a wealthy nation with decreasing forest resources; Fig 2B).

A significant positive correlation was observed between the rate of change of forest resources and the Human Development Index (UNDP) (Fig 2C; r = 0.60, p = 3.3e-11, see SI), showing expanding growing stock in high HDI countries. In contrast, the growing stock decreased especially in the African region, where HDI was low. We report annual Δ GS, but in reality the variable is measured as an average annual change between end-points of a multi-year period. Therefore, the regression is shown to GDP and HDI, rather than to their annual rates of change.

## Environmental changes and direct land-use impacts

Potapov et al. [12] estimated that intact forest landscapes including the water ecosystems within the forest landscape cover only 13.1 million km^2^ of the Earth surface. Direct human influence according to their study affects 42.8 million km^2^ of forest landscapes. Global croplands cover an estimated 14.7, and pastures 34.5 million km^2^, respectively [22]. Hence the environmental changes alone affect intact forest landscapes (13.1 Mkm^2^), while combinations of environmental changes and direct human influences affect a much larger area of the global terrestrial ecosystems (42.8 + 14.7 + 34.5 = 92 Mkm^2^). In most nations, direct land management affects a majority of the terrestrial ecosystems. Within exceptional regions of the World, where high-quality time series exist, research shows that land use policies and practices have affected deforestation and forest degradation for a long time [23,24].

Krausmann et al. [25] reported that from 1910 to 2005 the human population has grown fourfold and global economic output 17-fold, while the global human appropriation of net primary production (HANPP) has only doubled. However, large regional differences prevail. Unfortunately, deforestation continues at biologically rich forests [26]. The new expanding forests are biologically less diverse, especially where they consist of planted monocultures. Full impacts of the rapidly growing international trade are difficult to assess. Given the recent advances of forest transitions, the World can to approach a peak of agricultural land, implying prospects for the end of global deforestation [19].

New scientific methods of forest inventory have been developed this century. Changes in canopy cover are recorded from remote sensing data and have been made globally available wall-to-wall [27]. The results derived from remote sensing are reliable, if the focus is on abrupt losses of canopy cover in forest disturbances (logging, forest fire or storm damage). Estimating subtle incremental changes of the growing stock or biomass is more difficult, because the stem increment mainly occurs underneath a closed canopy. The rate of change of root biomass remains obscured from both space and land surface and must be estimated relative to changes in the above ground biomass.

**Fig 3:**
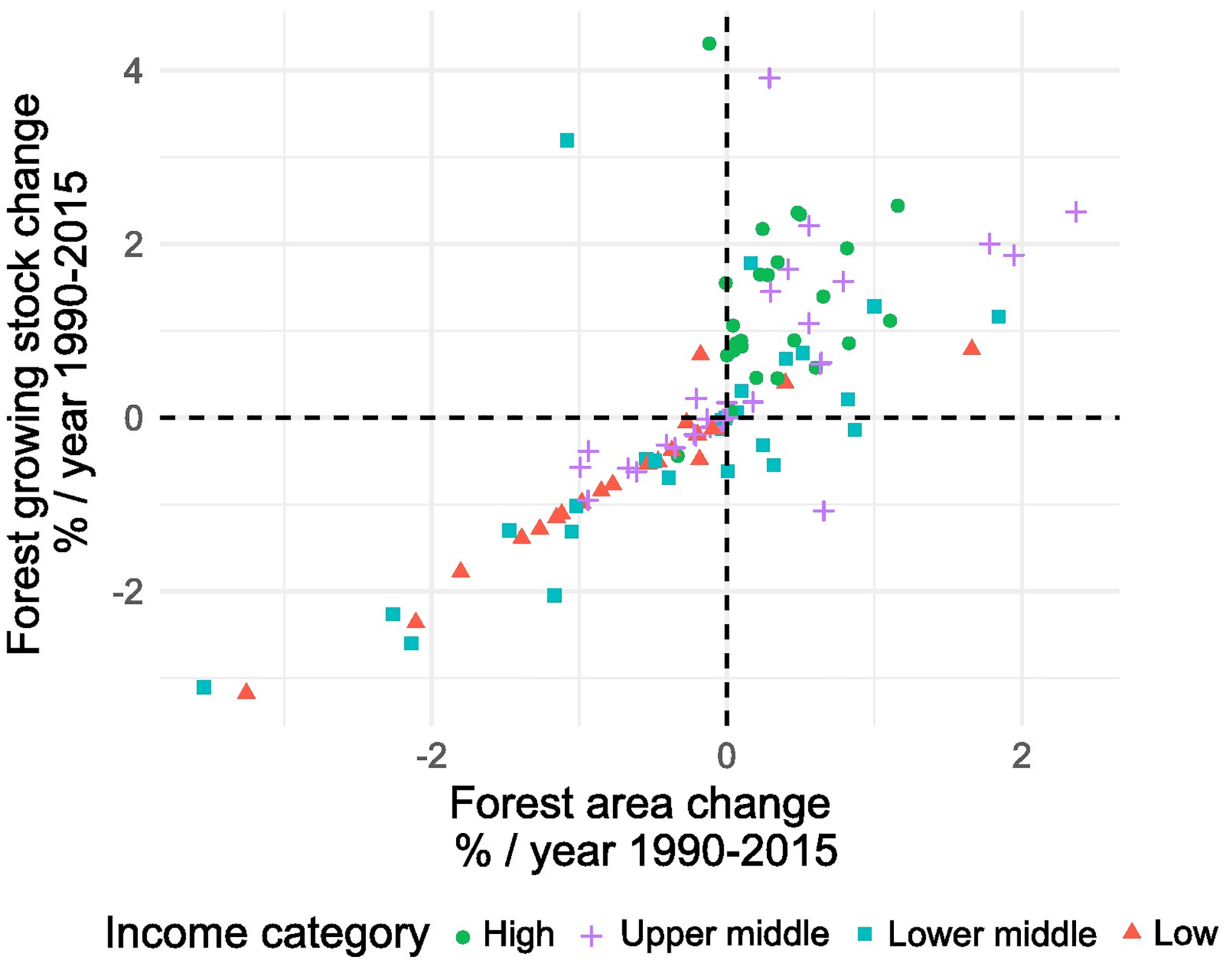
Percentage change of forest growing stock and forest area 1990–2015. The point colors represent the World Bank Income categories.

Low-income countries possess limited scientific capacity for land and forest inventory. Our data suggest that low-income countries tend to rely on remote sensing for their growing stock statistics (Fig 3). The positive and tight correlation between changes of growing stock and forest area indicate that growing stock has been estimated proportional to forest area. In other words, low-income countries lack measurements on forest degradation/densification. Such differences in research methods can explain some of the discrepancies between different global estimates of the terrestrial carbon trends [1,28].

In affluent societies, despite the positive forest trends, many sustainability challenges remain such as the reliance on fossil fuels [29]. A depletion of natural resources [30] and air, water and soil pollution [31] continue in many parts of the World. Large new changes in consumption and production patterns will be needed before the global populations can find a sustainable way of life.

Unfortunately, forest transition has been related to displacement of land use abroad in some cases [32]. Positive forest trends are rare in global biodiversity hot spots [33]. Some land management practices such as establishing timber plantations for industrial use have contributed to the expansion of woody biomass yet with detrimental impacts on quality of ecosystems [34]. The global international trade, which increased drastically during 1990–2015 [35], remains an important topic in future research analyzing the various drivers of the global greening and biomass change. Technologies, consumption patterns, priorities, and methods applied in farming, grazing and forest management must change before forests can flourish in all parts of the World.

We have shown historical lines of evidence on forest transitions and, for 1990–2015, a relationship between forest growing stock and HDI. Our research reports correlations, not causal relations. Human development can transform into well-being of forest ecosystems. This promotes carbon sequestration and preservation of the global biodiversity in the long term. Policy analyses must expand from focusing on individual projects on carbon capture, biodiversity conservation or farm management to inter-disciplinary analyses of harmonized well-being of people and forests.

Land use patterns change slowly but significantly over time [23]. Forest transition has taken place where agriculture improves, forestry becomes better organized and the demand for forest ecosystem services diversifies. In many low-income countries, deficient land management has overruled the potential of forest recovery. Regarding carbon sequestration in terrestrial ecosystems and social progress, synergies and co-benefits exist.

Our analysis shows how the deviations between countries in data quality restrict research in Earth sciences. For example, a new finding added approximately 9% to the earlier best estimates of the global forest are [16]. Some of such previously unknown forests may become attributed to forest expansion as the forest transition theory suggests. Unfortunately, empirical forest research has been deficient in tropical regions—the hot spots of global biodiversity [36,37]. Global scale monitoring of vegetation surfaces must be lifted to become a major priority area in World science.

We falsified the hypothesis that forest resources of the World expand because forest ecosystems respond primarily to environmental changes. Instead, we observed positive correlations of forest change with social, economic and technological progress much in accordance with the forest transition theory. The spatial patterns of forest trends correlated with social and economic attributes of nations rather than with environmental attributes. Our claim is that forest expansion [13], greening [2] and carbon sequestration [1] of the present magnitude would not have been possible in absence of economic, social and technological improvements.

## Supporting information

S1 Supporting Information (PDF)
