# Supplementary material for: Forest resources of nations in relation to human well-being
Source: PLoS One. 2018 May 14;13(5):e0196248. doi: 10.1371/journal.pone.0196248 (PMC5951536; doi:10.1371/journal.pone.0196248)
Supplement: S1 Supporting Information — (PDF) [file pone.0196248.s001.pdf]

Supporting information for

Forest resources of nations in relation to human well-being

Pekka E. Kauppi, Vilma Sandström & Antti Lipponen

correspondence to: pekka.kauppi@helsinki.fi

This PDF file includes:

Materials and Methods

Figs. S1 and S2

Table S1

Movie S1: Temperature anomalies arranged by country 1900 – 2016; © Antti Lipponen

Materials and Methods

We collected and used five sets of data.

Forest transitions

We updated the global map of forest transitions. These data refer to the national forest area. The main source was [1] with updates and additional findings for: Baltic States, Ukraine, Belarus and European Russia [2-5]; Colombia [6]; Costa Rica, El Salvador, Honduras, Panama [7]; Cuba, Puerto Rico, and the Dominican Republic [8]; Germany [9]; Italy [10,11]; Mexico [12-14]; Romania [15]; Spain [16,17]; Thailand [18]; Uruguay [19]; USA [20]; Venezuela [21].

Trends of forest growing stock

We compiled FAO data on changes in the forest growing stock from 1990 to 2015 [22]. The growing stock includes the stem volume of live trees from ground level up to a top diameter of 0 cm, excluding branches [23]. Whole-tree biomass can be estimated from stem data knowing that each tree is bound to have a photosynthesizing canopy and also a root system; and the ratio of dry biomass to stem volume (kg/liter) in woody material is within the range of 0.4 to 0.8. The carbon stock is estimated knowing that about 50% of dry woody biomass consists of carbon [20]. Changes of growing stock, 1990 to 2015, were related to three additional sets of data, which quantified climate warming, income level and Human Development Index, nation by nation.

Climate warming

The temperature changes (1990 – 2015) were estimated from the Berkeley Earth dataset [24]. The average temperature of Berkeley Earth monthly 1 x 1 degree latitude-longitude grid were estimated to compute the temperature changes by country. Country masks based on the Natural Earth dataset (large scale data, 1:10m) were applied. Overseas territories were excluded before computations and the temperature change of only the mainland area of countries were assessed. The original dataset was converted to a grid with resolution of 0.1 x 0.1 degrees using the nearest neighbor interpolation before computing the average temperature. This re-gridding was carried out to improve the

accuracy of country mask especially for small countries. We first computed the yearly average temperatures for each pixel. The annual average temperature for each country was computed utilizing the country mask. Finally, the temperature change was computed as the five-year average for the periods 1986-1990 and 2011-2015. The temperature change 1990-2015 was computed as the difference between these two periods. Long-term temperature changes in nations were demonstrated with a video (Temperature anomalies arranged by country 1900 – 2016; © Antti Lipponen; SI, p. n).

Income

World Bank data for the year 2013 on average income were used [25] noting categories low-income, lower-middle income, upper-middle income and high-income; Gross National Income, GNI per capita. The data refer to Gross Domestic Product, GDP, purchasing power parity PPP. The income ranges in terms of US$ were as follows: low-income: < 1,005; lower-middle income: > 1,006 < 3,975; higher-middle income: > 3,976 < 12,275; high-income: > 12,275.

Human Development Index

Since 1990, United Nations Development Programme (UNDP) has estimated and published Human Development Index (HDI) by nations. This index responds to statistics on life expectancy, education and other essential attributers of human well-being, many of which do not relate strictly to income per capita. The index adds a social dimension to analyses of the human well-being. We applied HDIs published for the year 2010 [26].

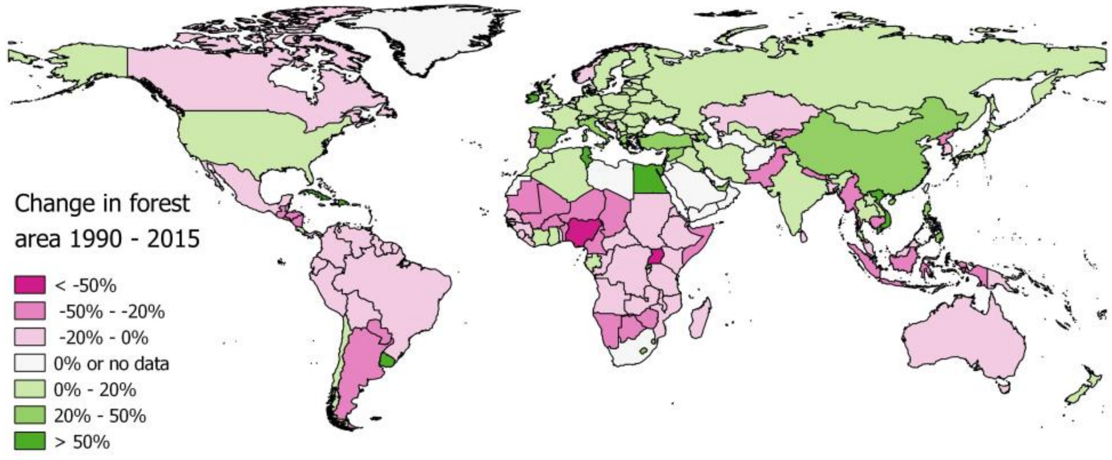

Fig. S1. Change of forest area during 1990-2015.

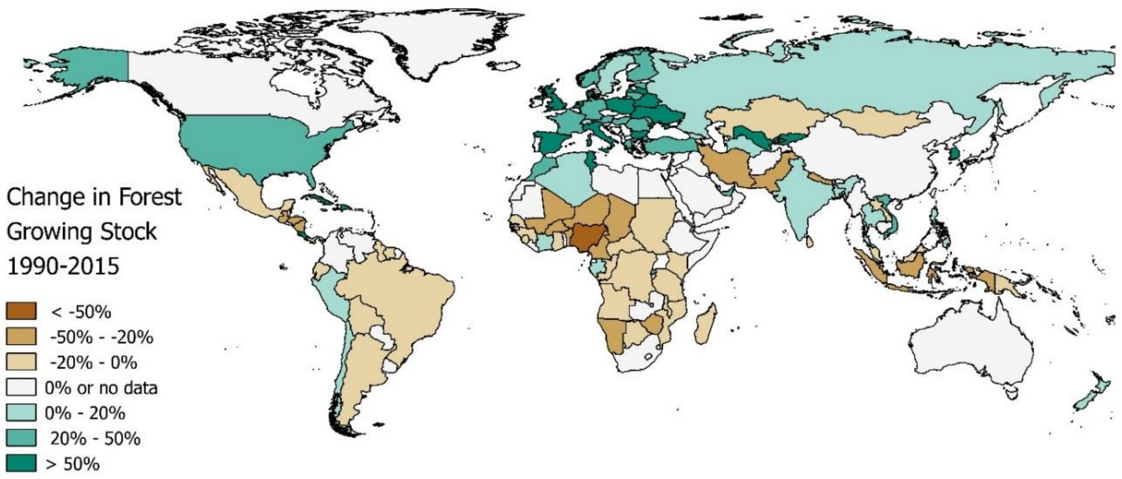

Fig. S2 Change of forest growing stock volume during 1990-2015.

Table S1.

Countries included and the data used in the analysis: country code, country name, region, income category, annual change of growing stock 1990-2015, GDP per capita (ppp) mean 2010-2015, HDI mean 2010-2015 and the temperature change 1990-2015.

Country	Income group	Forest growing stock 2015	Annual change of growing stock 1990-2015 (%)	GDP per capita (PPP) 2010-2015	HDI 2010–2015	Temp. change 1990–2015
Algeria	UM	89	0.63	13589	0.74	0.53
Angola	UM	2241	-0.21	6238	0.52	0.20
Argentina	UM	2667	-0.58	19640	0.82	0.28
Armenia	LM	41	-0.13	7724	0.74	0.93
Austria	H	1155	0.88	46356	0.89	1.15
Bangladesh	L	86	0.72	2854	0.57	0.13
Belarus	UM	1669	1.71	17732	0.79	0.98
Belize	UM	222	-0.58	8156	0.70	0.44
Benin	L	152	-1.15	1953	0.47	0.40
Bolivia (Plurinational State of)	LM	4133	-0.48	6119	0.66	0.52
Botswana	UM	725	-0.39	15114	0.69	0.03
Brazil	UM	96745	-0.32	15398	0.74	0.61
Brunei Darussalam	H	73	-0.44	81045	0.86	0.30
Bulgaria	UM	699	2.21	16525	0.78	0.96
Burkina Faso	L	225	-0.98	1543	0.39	0.40
Burundi	L	33	-0.49	796	0.40	0.40

Cameroon	LM	5802	-1.02	2873	0.50	0.37
Central African Republic	L	3751	-0.13	777	0.36	0.36
Chad	L	89	-1.29	2032	0.39	0.48
Chile	H	3316	0.57	21609	0.84	0.29
Congo	LM	4523	-0.07	5484	0.58	0.28
Costa Rica	UM	696	3.91	14305	0.77	0.29
Côte d'Ivoire	LM	2626	0.06	2928	0.46	0.29
Cuba	UM	213	2.00	NA	0.78	0.30
Cyprus	H	11	1.64	31833	0.85	0.96
Democratic Republic of the Congo	L	35115	-0.20	694	0.42	0.32
Denmark	H	120	2.36	45967	0.92	0.73
Dominican Republic	UM	119	2.37	12296	0.71	0.46
Ecuador	UM	3013	-0.63	10603	0.73	0.34
Estonia	H	476	0.77	26186	0.86	1.09
Finland	H	2320	0.85	40866	0.89	1.41
France	H	2935	1.39	38669	0.89	0.67
Gabon	UM	5405	0.18	16662	0.68	0.21
Gambia	L	18	0.39	1626	0.45	0.34
Georgia	LM	455	0.30	8218	0.76	1.01
Germany	H	3663	1.06	44302	0.92	0.85
Ghana	LM	378	-0.55	3717	0.57	0.34
Greece	H	193	0.85	26419	0.86	0.91
Guatemala	LM	461	-2.05	7165	0.62	0.41
Guinea	L	492	-0.53	1243	0.40	0.32
Guinea-Bissau	L	59	-0.51	1454	0.42	0.27
Guyana	LM	2991	-0.03	6676	0.63	0.56
Honduras	LM	556	-2.27	4253	0.62	0.44

Hungary	UM	377	1.08	23915	0.83	1.20
India	LM	5167	0.68	5154	0.60	0.11
Indonesia	LM	10227	-1.31	9680	0.68	0.23
Iran (Islamic Republic of)	UM	394	-1.08	17109	0.76	0.80
Italy	H	1385	1.95	36223	0.88	0.87
Jamaica	UM	52	-0.11	8253	0.73	0.19
Kazakhstan	UM	364	-0.02	22837	0.78	0.46
Kenya	L	836	-0.06	2721	0.54	0.52
Kyrgyzstan	LM	48	3.19	3101	0.65	0.61
Lao People's Democratic Republic	LM	920	-0.32	4825	0.57	0.42
Latvia	H	665	1.65	21690	0.82	0.95
Lithuania	H	515	0.89	25246	0.84	0.89
Madagascar	L	2133	-0.38	1407	0.51	0.42
Malawi	L	345	-0.85	1088	0.46	0.30
Malaysia	UM	5034	-0.05	23606	0.78	0.32
Mali	L	93	-1.39	1902	0.42	0.37
Mexico	UM	4727	-0.20	16406	0.75	0.64
Mongolia	LM	1406	-0.62	10267	0.72	0.40
Morocco	LM	154	0.74	7061	0.63	0.51
Mozambique	L	1377	-0.54	1044	0.41	0.25
Namibia	UM	166	-0.95	9404	0.63	0.00
Nepal	L	647	-1.11	2199	0.55	0.12
Netherlands	H	81	1.79	47431	0.92	0.73
New Zealand	H	3975	0.46	34714	0.91	0.04
Nicaragua	LM	482	-1.30	4636	0.63	0.36
Niger	L	11	-2.36	881	0.34	0.65
Nigeria	LM	936	-3.11	5571	0.52	0.50
Norway	H	1157	1.55	63351	0.94	0.88
Pakistan	LM	135	-2.60	4566	0.54	0.24
Panama	UM	751	-0.35	18872	0.77	0.34

Papua New Guinea	LM	5195	-0.01	NA	0.51	0.10
Peru	UM	8891	0.22	11312	0.73	0.46
Philippines	LM	1298	0.21	6346	0.67	0.09
Poland	H	2540	2.17	24176	0.84	0.90
Republic of Korea	H	918	4.31	32395	0.89	0.28
Republic of Moldova	LM	50	1.28	4502	0.69	1.02
Romania	UM	1930	1.45	19501	0.80	0.95
Russian Federation	H	81488	0.07	24092	0.80	1.10
Rwanda	L	85	0.78	1567	0.48	0.39
Senegal	LM	308	-0.50	2262	0.48	0.42
Sierra Leone	L	121	-0.14	1461	0.41	0.27
South Africa	UM	670	0.00	12637	0.65	0.19
Spain	H	1212	2.44	32832	0.88	0.43
Sri Lanka	LM	46	-0.69	10227	0.76	0.29
Suriname	UM	3816	-0.03	15341	0.72	0.57
Swaziland	LM	19	-0.14	7901	0.54	0.17
Sweden	H	2989	0.71	45008	0.91	1.05
Switzerland	H	442	0.45	58327	0.94	0.86
Thailand	UM	1506	0.62	14771	0.73	0.40
Tunisia	UM	27	1.87	10777	0.72	0.54
Turkey	UM	1506	1.57	21206	0.76	0.91
Turkmenistan	UM	15	0.17	13029	0.68	0.44
Uganda	L	92	-3.18	1676	0.48	0.46
Ukraine	LM	2196	1.78	8281	0.74	1.24
United Arab Emirates	H	16	1.11	63041	0.83	1.21
United Kingdom	H	652	2.34	38528	0.90	0.62

United Republic of Tanzania	L	3333	-0.77	2347	0.51	0.41
United States of America	H	40699	0.82	52201	0.92	0.41
Viet Nam	LM	878	1.16	5184	0.67	0.29
Zimbabwe	L	540	-1.78	1828	0.49	0.11

Movie S1. Temperature anomalies arranged by country 1900 - 2016, © Antti Lipponen; see:

https://www.youtube.com/watch?v=jWFxoAhic1oS1

References

1. Meyfroidt P, Lambin EF. Global forest transition: prospects for an end to deforestation. Annu. Rev. Environ. Resour. 2011;36: 343-371.
2. Taff GN, Müller D, Kuemmerle T, Ozdeneral E, Walsh SJ. Reforestation in Central and Eastern Europe after the breakdown of socialism. In: Anonymous Reforesting Landscapes : Springer; 2009. pp. 121-147.
3. Ruskule A, Nikodemus O, Kasparinska Z, Kasparinskis R, Brūmelis G. Patterns of afforestation on abandoned agriculture land in Latvia. Agrofor Syst. 2012;85: 215-231.
4. Vanwambeke SO, Meyfroidt P, Nikodemus O. From USSR to EU: 20 years of rural landscape changes in Vidzeme, Latvia. Landscape Urban Plann. 2012;105: 241-249.
5. Kuemmerle T, Kaplan JO, Prishchepov AV, Rylsky I, Chaskovskyy O, Tikunov VS, et al. Forest transitions in Eastern Europe and their effects on carbon budgets. Global Change Biol. 2015;21: 3049-3061.

6. Sánchez-Cuervo AM, Aide TM, Clark ML, Etter A. Land cover change in Colombia: surprising forest recovery trends between 2001 and 2010. PLoS One. 2012;7: e43943.
7. Redo DJ, Grau HR, Aide TM, Clark ML. Asymmetric forest transition driven by the interaction of socioeconomic development and environmental heterogeneity in Central America. Proc. Natl. Acad. Sci. U.S.A. 2012;109: 8839-8844.
8. Álvarez-Berríos NL, Redo DJ, Aide TM, Clark ML, Grau R. Land Change in the Greater Antilles between 2001 and 2010. Land. 2013;2: 81-107.
9. Niedertscheider M, Kuemmerle T, Mller D, Erb K. Exploring the effects of drastic institutional and socio-economic changes on land system dynamics in Germany between 1883 and 2007. Global Environ Change. 2014;28: 98-108.
10. Moretti V, Ferrara A, Colantoni A, Carlucci M, Salvati L. Forest transition and changes in the socio-economic structure of a developed country: a long-term analysis. Modern Economy. 2014;2014.
11. Niedertscheider M, Erb K. Land system change in Italy from 1884 to 2007: Analysing the North–South divergence on the basis of an integrated indicator framework. Land Use Policy. 2014;39: 366-375.
12. Bonilla-Moheno M, Aide TM, Clark ML. The influence of socioeconomic, environmental, and demographic factors on municipality-scale land-cover change in Mexico. Reg. Environ. Chang. 2012;12: 543-557.
13. Bonilla-Moheno M, Redo DJ, Aide TM, Clark ML, Grau HR. Vegetation change and land tenure in Mexico: A country-wide analysis. Land Use Policy. 2013;30: 355-364.
14. García-Barrios L, Galván-Miyoshi YM, Valdivieso-Pérez IA, Masera OR, Bocco G, Vandermeer J. Neotropical forest conservation, agricultural intensification, and rural out-migration: the Mexican experience. Bioscience. 2009;59: 863-873.
15. Olofsson P, Kuemmerle T, Griffiths P, Knorn J, Baccini A, Gancz V, et al. Carbon implications of forest restitution in post-socialist Romania. Environ. Res. Lett. 2011;6: 045202.

16. Marey-Pérez MF, Rodríguez-Vicente V. Forest transition in Northern Spain: local responses on large-scale programmes of field-afforestation. Land Use Policy. 2009;26: 139-156.
17. Valbuena-Carabaña M, López de Heredia U, Fuentes-Utrilla P, González-Doncel I, Gil L. Historical and recent changes in the Spanish forests: a socio-economic process. Rev Palaeobot Palynol. 2010;162: 492-506.
18. Leblond J, Pham TH. Recent forest expansion in Thailand: a methodological artifact? J. Land Use Sci. 2014;9: 211-241.
19. Redo DJ, Aide TM, Clark ML, Andrade-Nunez MJ. Impacts of internal and external policies on land change in Uruguay, 2001–2009. Environ Conserv. 2012;39: 122-131.
20. Kauppi PE, Ausubel JH, Fang J, Mather AS, Sedjo RA, Waggoner PE. Returning forests analyzed with the forest identity. Proc. Natl. Acad. Sci. U.S.A. 2006;103: 17574-17579.
21. Aide TM, Clark ML, Grau HR, López-Carr D, Levy MA, Redo D, et al. Deforestation and reforestation of Latin America and the Caribbean (2001–2010). Biotropica. 2013;45: 262-271.
22. MacDicken K, Jonsson Ő, Piňa L, Maulo S, Adikari Y, Garzuglia M, et al. Global Forest Resources Assessment 2015: How have the world's forests changed? Food and agriculture organization of the United Nations, FAO. 2016.
23. FRA 2015 Terms and Definitions. 2012; Forest Resources Assessment Working Paper 180. Food and agriculture organization of the United Nations, FAO.
24. Rohde R, Muller R, Jacobsen R, Perlmutter S, Rosenfeld A, Wurtele J, et al. Berkeley Earth Temperature Averaging Process, Geoinfor. Geostat.: An Overview 1: 2. of. 2013;13: 20-100.
25. The World Bank. World Development Indicators. Available: http://data.worldbank.org/.
26. Jahan S. The Human Development Report 2016 'Human Development for Everyone'. United Nations Development Programme (UNDP), New York, USA. 2016.
